# Time-domain Tollens reaction: synthesising silver nanoparticles with the formaldehyde clock†‡

**DOI:** 10.1039/d3na00121k

**Published:** 2023-03-16

**Authors:** Ronny Kürsteiner, Maximilian Ritter, Alla Sologubenko, Laura Stricker, Guido Panzarasa

**Affiliations:** a Institute for Building Materials, Department of Civil, Environmental and Geomatic Engineering, ETH Zürich Laura-Hezner-Weg 7 8093 Zürich Switzerland guidop@ethz.ch; b Scientific Center for Light and Electron Microscopy (ScopeM), ETH Zürich Otto-Stern-Weg 3 8093 Zürich Switzerland

## Abstract

The addition of silver(i) ions to the methylene glycol–sulphite (MGS) clock reaction results in the sudden formation of metallic silver nanoparticles. Stable suspensions are obtained in the presence of poly(vinylpyrrolidone). The time delay before the appearance of the particles, as well as their size, decreases with the initial methylene glycol concentration while their monodispersity increases.

## Abstract 2

The methylene glycol–sulfite clock reaction enables the time-programmable synthesis of silver nanoparticles with tailorable size and monodispersity.

## Graphical abstract

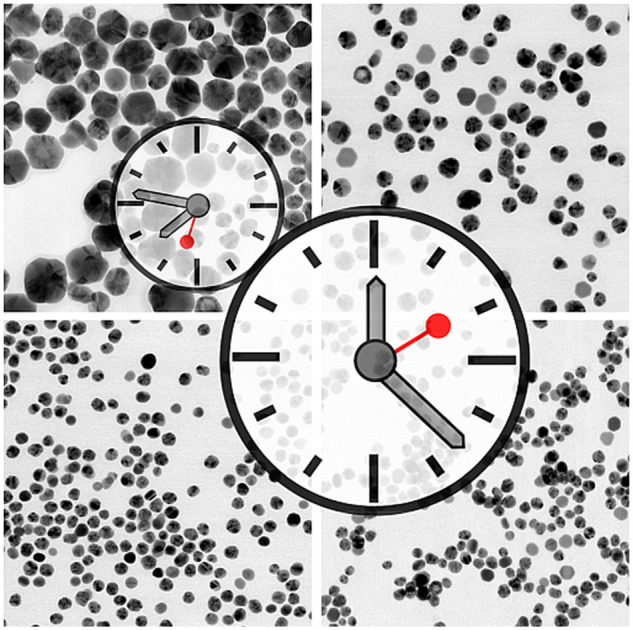

In 1882, B. Tollens presented an extensive study on the reduction of silver(i) (hydr)oxide to metallic silver by aldehydes, reactions of great analytical as well as synthetic value.^1,2^ In the classical Tollens reaction, the reagent is not silver (hydr)oxide but its water-soluble complex with ammonia. Since then, the Tollens reaction has been used for depositing thin films and for nanoparticle synthesis, enabling a variety of applications ranging from electrical conductors to catalysis, from antimicrobial agents to plasmonics.^3–7^

Our research concentrates on the materials science applications of clock reactions,^8–15^ and during our studies we realised that the methylene glycol–sulphite (MGS) reaction, also termed “formaldehyde clock”, could be suitable for the reduction of silver(i) ions to metallic silver, in close analogy with the classical Tollens reaction. The MGS reaction is a well-known pH clock featuring a sudden pH increase, from acidic (pH_initial_ ≈ 5.5) to alkaline (pH_final_ ≈ 11), after a highly reproducible induction time which is a function of the initial reactant concentrations.^16,17^ The mechanism of the MGS clock is presented by eqn (1)–(4) in Scheme 1. Bisulphite HSO_3_^−^ and sulphite SO_3_^2−^ species are in a dynamic equilibrium (eqn (1)), resulting in the formation of a pH-buffering system. Methylene glycol MG CH_2_(OH)_2_ spontaneously dehydrates into formaldehyde CH_2_O (eqn (2)), which quickly reacts with sulphite generating hydroxymethanesulphonate HOCH_2_SO_3_^−^ and hydroxide ions OH^−^ (eqn (3)). However, while bisulphite is present, the base is scavenged with the formation of sulphite (eqn (4)) which feeds back into the system. As a result, the pH remains fairly constant until the bisulphite buffer is depleted, after which it increases suddenly. The final amount of OH^−^ generated equals that of the initial sulphite concentration, while for a fixed SO_3_^2−^/HSO_3_^−^ ratio the induction time is directly proportional to the initial concentration of formaldehyde (which remains always in excess).

**Scheme 1:**
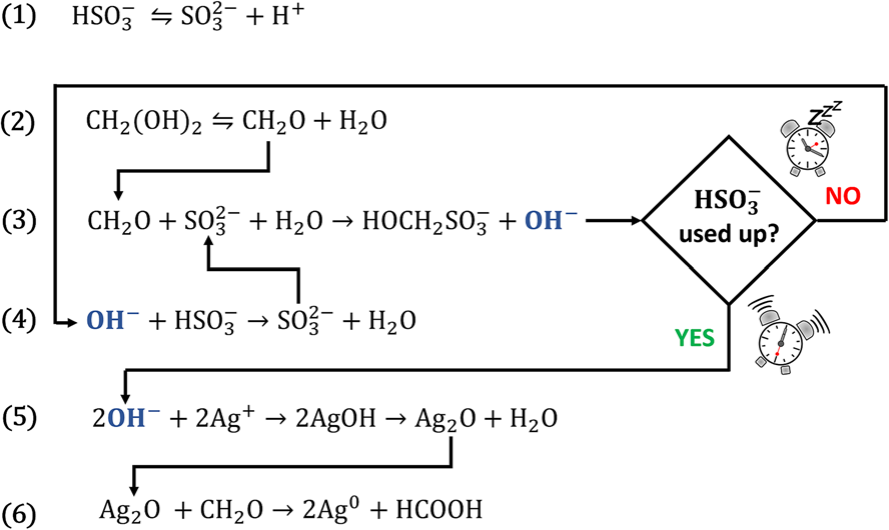
The mechanism of the MGS–Ag(i) system.

Recently, the application of the MGS clock for the synthesis of hybrid (*e.g.* the metal–organic framework ZIF-8)^18^ and inorganic (*e.g.* iron (hydr)oxides)^15^ materials has been demonstrated. In the present work, we applied the same principle to obtain silver nanoparticles. When silver(i) ions are added to the MGS reaction, silver(i) hydroxide AgOH precipitates after the clock sets off, then spontaneously dehydrates to form the oxide Ag_2_O (eqn (5)). The latter is then reduced to metallic silver by the formaldehyde, following the route of the Tollens reaction (eqn (6)).

For our investigations we chose to use the following initial reactant concentrations: [SO_3_^2−^]_0_ = 5 mM, [HSO_3_^−^]_0_ = 50 mM, and [MG]_0_ ranging from 100 to 500 mM. These conditions result in the generation of *ca.* 5 mM OH^−^, which is more than sufficient to react with 0.5 mM silver(i) ions (added as nitrate). Reactions were carried out at room temperature (23 ± 1 °C). Silver(i) is soluble in presence of sulphite in excess due to the formation of a complex, which is stable in the reaction conditions.^19^

The initially transparent and colourless reaction mixture transforms into a greyish suspension. Later on, it forms black agglomerates, readily dispersible by sonication (inset of Fig. 1A, S1 and Movie S1‡). A qualitative test with acetic acid§§Silver (hydr)oxide is soluble in acetic acid, while metallic silver is not. confirmed that the final product is metallic silver and not silver (hydr)oxide. UV-vis spectra acquired as a function of time (Fig. 1A) demonstrated a gradual increase of absorbance at the beginning, followed by prompt evolution of the curves to exhibit a distinct broad peak centred at 418 nm. This feature, along with the absorption tail at longer wavelengths, can be associated to the surface plasmon resonance (SPR) of aggregated silver nanoparticles. Scanning transmission electron microscopy (STEM) data (Fig. 1B) demonstrated that the product consists of aggregated spherical and elongated particles 40–60 nm in size. In addition, yet another silver-free component is detected in the material. Analyses of the selected area electron diffraction patterns (SAED, Fig. 1D) evidenced the presence of metallic silver in the product and suggests that the silver-free phase could be a sodium hydroxymethanesulphonate hydrate (see also Fig. S2‡). The energy dispersive X-ray spectroscopy (EDS) in the spectrum imaging (SI) mode of STEM (Fig. S3‡) indeed showed the presence of sulphur in the material, indicating that hydroxymethanesulphonate had likely adsorbed on the silver nanoparticle surfaces.

**Fig. 1:**
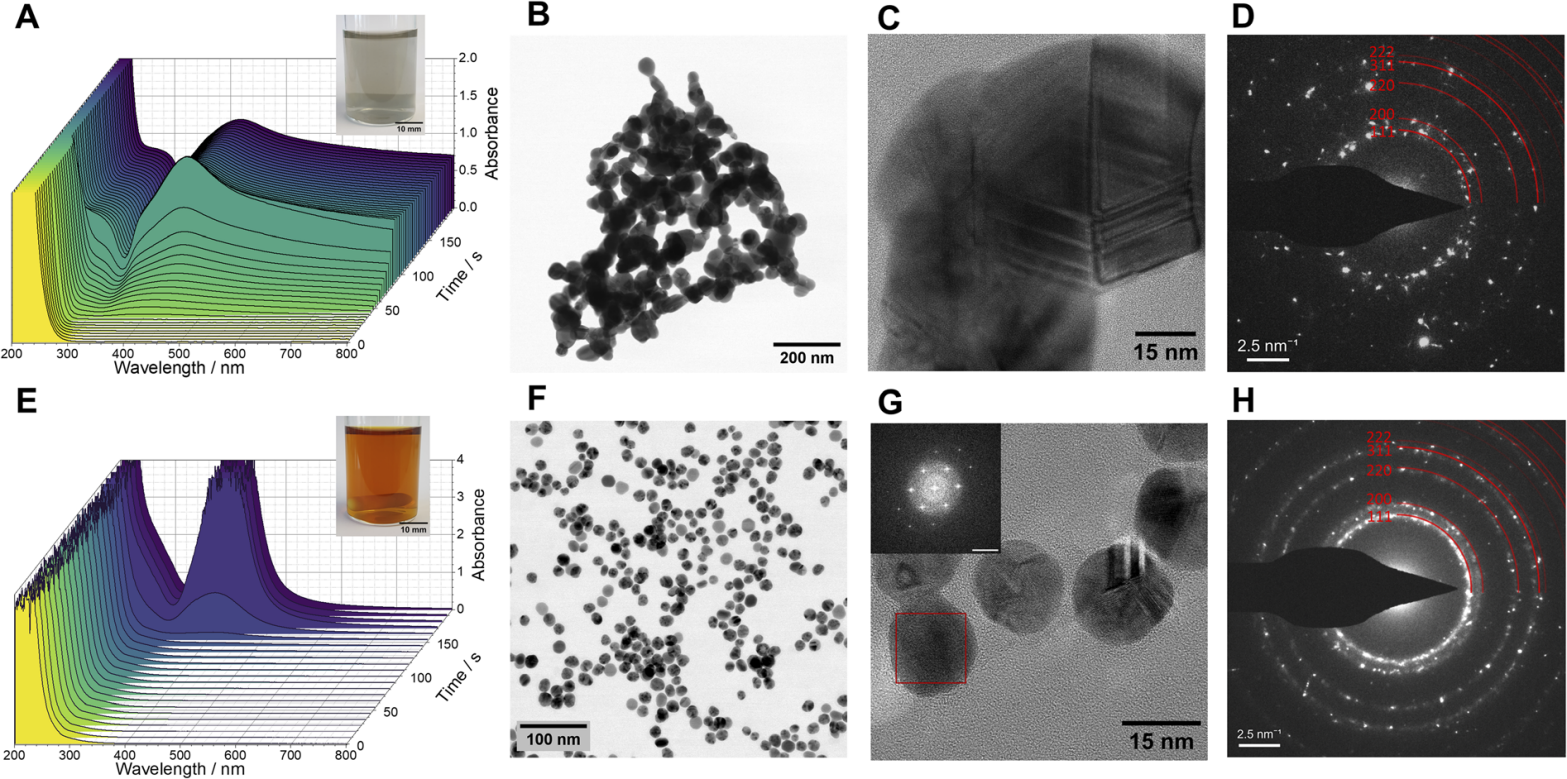
Formation of silver nanoparticles in the MGS–Ag(i) system ([MG]_0_ = 200 mM) without (A–D) and with (E–H) 1% poly(vinylpyrrolidone) PVP 10 kDa. (A and E) UV-vis spectra showing the evolution over time of the optical properties. The insets show pictures of the reacted mixtures. (B and F) Bright-field STEM micrographs, (C and G) high-resolution TEM micrographs. The inset in (G) shows the fast-Fourier transform (FFT) of an un-twinned silver nanoparticle (red box) in [111] zone axis (scale bar: 5 nm^−1^). (D and H) Selected area electron diffractograms of the resulting silver nanoparticles, evidencing their fcc-structure.

To prevent particle aggregation, we added 1%_m/v_ poly(*N*-vinylpyrrolidone) (PVP, avg. *M*_w_ 10 kDa), a commonly used steric stabiliser for silver nanoparticles,^20^ to the MGS–Ag(i) system. In presence of PVP, the initially colourless reaction mixture evolved over time into a homogeneous amber-coloured and transparent suspension (inset of Fig. 1E and Movie S2‡). The induction time of the pH clock was not significantly affected by the presence of PVP (Fig. S4‡). As seen from the UV-vis spectrum as a function of time (Fig. 1E), an intense and relatively narrow peak, centred at 405 nm, developed rapidly after some latent period. This peak is characteristic for the surface plasmon resonance of well-dispersed, spherical, and small (<100 nm in diameter) silver nanoparticles.^21^ Indeed, the high-resolution TEM micrographs revealed a highly uniform spherical morphology of the particles, 15–20 nm in size (Fig. 1G), displaying the characteristic contrast of nano-sized twins in silver nanoparticles. The analyses of SAED patterns (Fig. 1H) and fast Fourier transforms (FFTs) of the HR-TEM images also confirmed that the product is metallic silver. We also tried reducing the concentration of PVP to 0.1 and 0.5%_m/v_, but this resulted in more polydisperse particles. Although the UV-vis spectra of the particle suspensions obtained with 0.5% and 1% PVP were virtually identical (Fig. S5‡), more detailed information was revealed by electron microscopy. The suspension made with 0.5% PVP exhibited significantly higher polydispersity (Fig. S6‡). Increasing the concentration of silver(i) ions (to 0.75 and 1 mM) in the reaction mixture led to particles with increased polydispersity, as pointed out by the UV-vis spectra (Fig. S7‡) and electron micrographs (Fig. S8‡).

PVP is known to form complexes with Ag(i) ions and to be able to reduce them to metallic silver thanks to its end-groups.^22^ To better understand its role in the MGS–Ag(i) system, we performed additional control experiments. As a result, we established that in the presence of PVP, no silver (hydr)oxide forms when 5 mM NaOH is added to 0.5 mM Ag(i). Instead, an amber-coloured suspension containing much more polydisperse Ag-particles with sizes ranging from 2 to 50 nm forms (Fig. S9 and Movie S3‡). Repeating this experiment with 200 mM of MG, we obtain a dark brown suspension which, within 20 min, evolves into a dichroic suspension (amber-coloured in transmission, hazy brown/grey in reflection, Movie S4‡). It contains roughly spherical, metallic silver particles up to ∼100 nm in size with a broad size distribution (Fig. S10‡). These control experiments showed the superiority of the MGS clock-based approach in terms of particle homogeneity and monodispersity.

Moreover, we investigated the effect of the initial MG concentration on the behaviour of the MGS–Ag(i)–PVP system, simultaneously measuring the time-evolution of the pH value (indicative of the MGS clock reaction) and the evolution of absorbance (indicative of the formation of silver particles). Varying the [MG]_0_ in the MGS clock allows to tune the induction time of the pH increase. For the conditions used here, this amounted to about 8 s ([MG]_0_ = 500 mM) to 50 s ([MG]_0_ = 100 mM) time periods. Although both, pH (Fig. 2A) and absorbance (measured at 400 nm, Fig. 2B), show a sharp increase after an induction time, there is a significant time lag between them. A closer inspection of the absorbance profiles reveals a direct correlation between the two in the form of a small increase in absorbance (Fig. S11‡), which occurs at the moment of the pH increase. We attribute this behaviour to the formation of AgOH (Scheme 1, eqn (5)). The evolution of absorbance over time can be as fast as 27 s ([MG]_0_ = 500 mM) or can take up to 1200 s ([MG]_0_ = 100 mM) and is clearly auto-accelerating, a behaviour sometimes observed during the synthesis of silver nanoparticles with the Tollens reaction.^23,24^

**Fig. 2:**
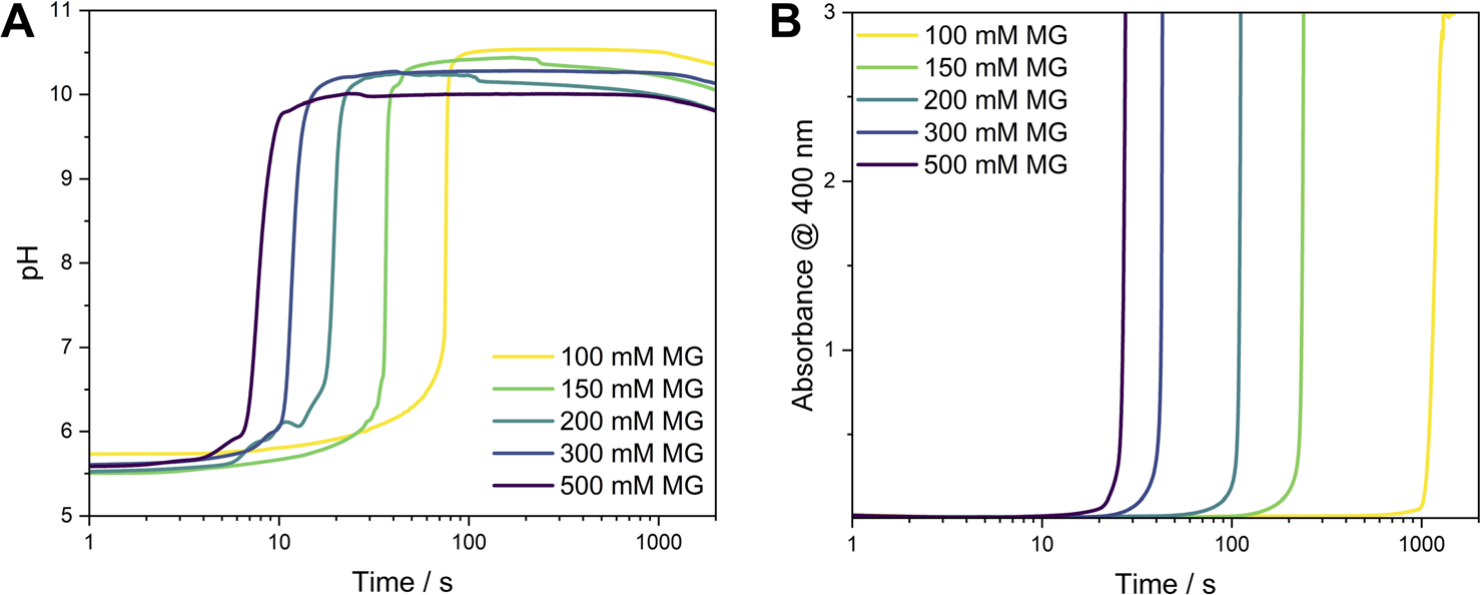
(A) Evolution of pH in the MGS–Ag(i)–PVP system as a function of time. The initial pH value is 5.6 ± 0.1. (B) The development of the Ag SPR peak as a function of time was monitored by the absorbance at 400 nm.

In addition to varying the induction time for the formation of silver nanoparticles, we found that varying [MG]_0_ also leads to different particle sizes. The UV-vis spectra of samples obtained for [MG]_0_ ≥ 150 mM (Fig. S12‡) showed a narrow SPR peak at ∼400 nm. For [MG]_0_ = 100 mM a significant broadening is observed, indicative of larger particles. Image analyses (Fig. 3) of the STEM micrographs (Fig. 3 and S18–S22‡) showed that the size distribution remains virtually constant for [MG]_0_ ≥ 200 mM, while for [MG]_0_ ≤ 150 mM significant broadening and a shift to larger particle sizes was observed.

**Fig. 3:**
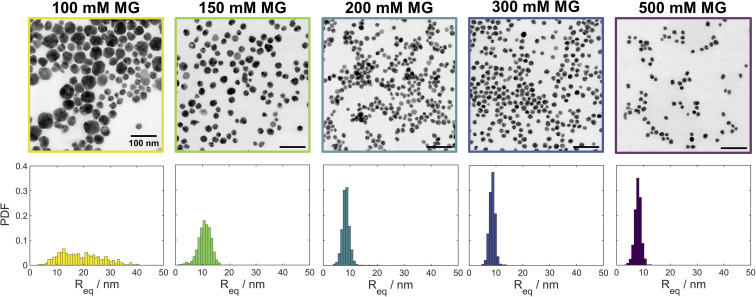
Bright field STEM micrographs and particle size distributions showing the effect of the initial methylene glycol concentration [MG]_0_ (indicated on top of each image) in the MGS–Ag(i)–PVP system on the morphology and size distribution of the resulting silver nanoparticles. The scale bar is 100 nm in all images. For the corresponding high-angle annular dark field (HAADF) and low-angle annular dark field (LAADF) micrographs see Fig. S13–S17.‡

In order to probe a larger number of particles for size analysis than is available *via* analysis of the STEM images, small angle X-ray scattering (SAXS) was performed on both “clocked” and control systems. The size distribution was simulated using Xenocs XSACT (Fig. 4 and S23‡) and the pair distance distribution function (PDDF, Fig. S24‡) was analysed using the ATSAS (Version 3.0.5) software package.^25^ The average particle size decreases with [MG]_0_, most likely due to a faster initial nucleation. The particle size distribution becomes narrower for the higher [MG]_0_, except in the case of [MG]_0_ = 500 mM, for which the slight broadening is detected and is attributed to the presence of larger particles. It is noteworthy to mention that the particle size distribution analyses from STEM micrographs and from the SAXS data are in good agreement.

**Fig. 4:**
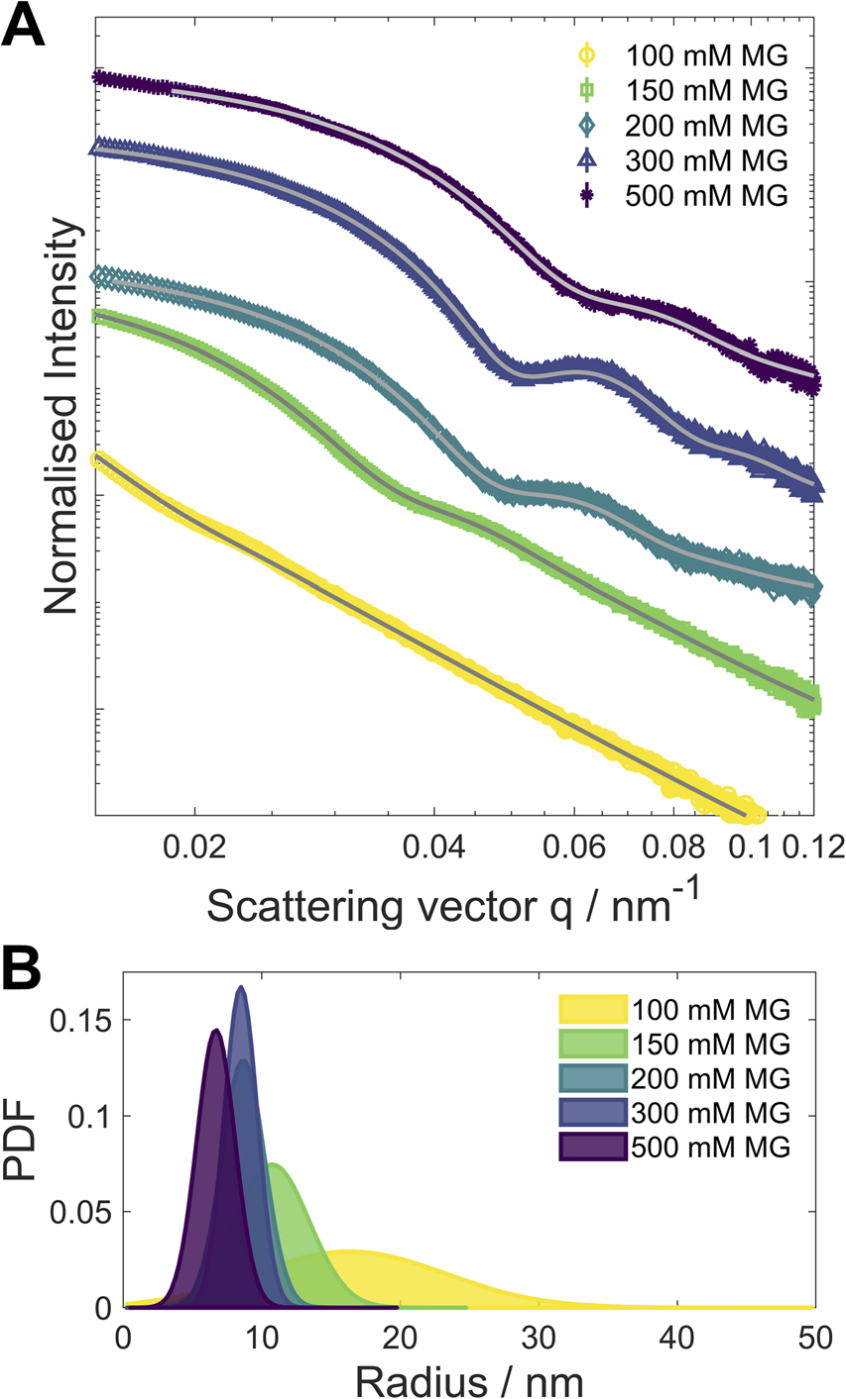
(A) SAXS scattering curves (symbols, normalised intensity plotted over the scattering vector *q*). The curves have been shifted vertically for improved readability. (B) Diameter distribution as determined from the SAXS scattering curves, the corresponding fits for the distributions are shown in (A) as grey lines.

## Conclusions

We demonstrated that the addition of silver(i) ions to the methylene glycol–sulphite (MGS) clock reaction results in the spontaneous formation of silver nanoparticles after an induction time. Together with the particle morphology, this induction time can be tuned by varying the initial MG concentration and by the addition of PVP. The base produced by the MGS clock results in the formation of silver (hydr)oxide, which in turn is reduced to metallic silver by the formaldehyde in excess, similar to what happens in the classical Tollens reaction. These results further show the usefulness of chemical clocks for the design of time-programmable autonomous self-assembly systems.

## Author contributions

Conceptualization: G. P.; formal analysis: L. S., R. K., M. R., A. S.; funding acquisition: G. P.; investigation: G. P., R. K., M. R., A. S.; methodology: G. P., R. K.; project administration: G. P.; supervision: G. P.; validation: G. P., R. K.; visualization: G. P., R. K.; writing – original draft: G. P., R. K.; writing – review and editing: G. P., R. K.

## Conflicts of interest

There are no conflicts to declare.

## Supplementary Material

NA-005-D3NA00121K-s001

NA-005-D3NA00121K-s002

NA-005-D3NA00121K-s003

NA-005-D3NA00121K-s004

NA-005-D3NA00121K-s005
