# Supplementary material for: Time-domain Tollens reaction: synthesising silver nanoparticles with the formaldehyde clock
Source: Nanoscale Adv. 2023 Mar 16;5(8):2175–9. doi: 10.1039/d3na00121k (PMC10089108; doi:10.1039/d3na00121k)
Supplement: NA-005-D3NA00121K-s005 [file NA-005-D3NA00121K-s005.pdf]

Electronic Supplementary Material (ESI) for Nanoscale Advances.
This journal is © The Royal Society of Chemistry 2023

SUPPORTING INFORMATION

Time-Domain Tollens Reaction: Synthesising Silver Nanoparticles with the Formaldehyde Clock

Ronny Kürsteiner,[a] Maximilian Ritter,[a] Alla Sologubenko,[b] Laura Stricker,[a] and Guido Panzarasa*[a]

[a] Institute for Building Materials, Department of Civil, Environmental and Geomatic Engineering, ETH Zürich, Laura-Hezner-Weg 7, 8093 Zürich, Switzerland.

[b] Scientific Center for Light and Electron Microscopy (ScopeM), ETH Zürich, Otto-Stern-Weg 3, 8093 Zürich, Switzerland

*Corresponding author. E-mail: guidop@ethz.ch

Experimental

Reagents. Sodium sulphite (≥98%), sodium bisulphite (ACS reagent, mixture of $NaHSO_3$ and $Na_2S_2O_5$), sodium hydroxymethanesulphonate (formaldehyde-sodium bisulphite adduct, HMSNa, 95%), poly(vinylpyrrolidone) (average molecular weight 10 kDa), and silver nitrate were purchased from Sigma-Aldrich (Switzerland). Concentrated aqueous formaldehyde solution (37-41%, stabilized with 12% methanol) was purchased from Fisher Scientific (UK). Sodium hydroxide NaOH (Convol NORMADOSE 1N) were purchased from VWR (Czech Republic). Unless otherwise stated, all chemicals were of analytical or reagent grade purity and used as received. Water was purified using a MilliQ system (resistivity ≥18 MΩ).

pH Measurements. A Hanna Instruments (USA) HI5222-02 benchtop pH-meter was used together with a HI1330B glass body combination pH microelectrode from the same Company. The pH-meter was calibrated with standard buffer solutions (pH values: 1.670, 4.010, 7.010, 10.01, 12.45) before each set of analysis. The pH-electrode was cleaned after each analysis by repeated immersion in water, the excess water gently removed with hairless paper, and immediately immersed in the solution to analyse. The pH-meter was interfaced with a computer through the software HI92000 – 5.0.38 (Hanna Instruments, USA) to allow continuous recording of pH values with a time interval of 1 s.

UV-Vis spectroscopy. Time-resolved absorption measurements at fixed wavelength (400 nm) were performed with a UV-visible spectrophotometer (PerkinElmer LAMBDA 650) using optically-matched quartz cuvettes with an optical path of 1 cm and a temporal resolution of 1 s. The content of the cuvette was stirred (500 rpm) with a suitable magnetic bar. Full spectrum time-resolved absorption measurements were performed with a Cary 60 UV-visible spectrophotometer (Agilent) using optically-matched quartz cuvettes with an optical path of 1 cm and a spectral resolution of 1 nm (sample with PVP) or 5 nm (sample without PVP).

Analytical (scanning) transmission electron microscopy ((S)TEM) was performed on an FEI Talos F200X operated at 200 kV acceleration voltage. Elemental content distribution mapping was carried out by energy dispersive X-ray spectroscopy (EDS) STEM spectrum imaging with a windowless Bruker Super-X EDS system. The EDS

spectra were acquired up to 20 keV with a spectral resolution of 10 eV per channel and an acquisition time of 300 s. Selected-area electron diffraction (SAED) patterns were acquired with a 10 µm SAD aperture corresponding to a diameter of 200 nm in the object plane.

Image Analysis. The particle sizes are evaluated from the STEM images by using the FiJi image processing software [1]. The original grey-scale images are first converted into black-and-white images, with the native Threshold FiJi plugin. When required, the particles are separated from each other by applying a watershed immersion algorithm [2], by means of its implementation in the native FiJi Watershed plugin. The particles are then detected with the Analyze particles plugin, which also provides their area. The images are visually inspected to verify the quality of the detection. Finally, from the area A_i of the ith particle, we calculate its equivalent radius as (eq. S1):

$$R_{eq,i} = \sqrt{\frac{A_i}{\pi}} \quad \text{(S1)}$$

In Fig. 3, we plot the Probability Distribution Function (PDF) of the equivalent radii.

Small Angle X-Ray Scattering (SAXS). For the SAXS measurements, the as-synthesized silver suspensions (10 mL) were centrifuged for 30min at 9000RPM and redispersed in 500 µL of a 1% (w/v) PVP10 solution. The nanoparticle suspensions were injected into glass mark-tubes (Hilgenberg) with an outer diameter of 0.7 mm and a wall thickness of 0.01 mm and sealed using a two-component epoxy resin. The capillaries were inserted into a laboratory SAXS system (Xenocs Xeuss 3.0) and the scattering signal was recorded under vacuum using Cu Kα X-ray radiation ($\lambda Cu_{K\alpha}$ = 1.5419 Å) and a 2D detector (Dectris EIGER2 1M) positioned at a sample-to-detector distance of 1300 mm. The recorded 2D scattering signal was azimuthally integrated to obtain the 1D scattering signal (scattering intensity as a function of the scattering vector). The background scattering from the capillary and water was scaled and then subtracted from the recorded signals to obtain the scattering signal solely from the silver particles. All data handling was performed using Xenocs XSACT software [3]. The size distribution was simulated using the dilute spheres tool in the XSACT software. The pair distance distribution function (PDDF) was analysed using the ATSAS (Version 3.0.5) software package [4].

X-Ray powder diffraction (XRPD). A PANalytical X'Pert PRO MPD diffracrometer using monochromated Cu $K_{\alpha1}$ radiation (40 kV, 45 mA) in Bragg–Brentano geometry was used. Sodium hydroxymethane sulphonate (CAS 870-72-4) was recrystallized from water and dried under vacuum at room temperature. The recrystallized product was confirmed to be the mono hydrate by thermogravimetric analysis. The dried sample was ground using an agate pestle and mortar and deposited on a silicon low-background sample holder. The diffractogram was obtained from 5° to 80° with an acquisition time of 75 min and a step size of 0.0334°.

Experimental protocols. A sulphite-bisulphite stock solution, containing 14 mM sulphite and 140 mM bisulphite, was prepared by dissolving 70 mg of sodium sulphite and 578 mg of sodium bisulphite in 40 mL of water. This solution was made fresh before use and discarded after two hours. A 6.5 M methylene glycol (MG) stock solution was prepared by diluting one part of the concentrated (ca. 13 M) aqueous solution with one part of water, and aged overnight before use. A 50 mM silver(I) stock solution was prepared by dissolving 84.9 mg $AgNO_3$ in 10 mL of water. All stock solutions were stored at room temperature avoiding direct light.

In a typical reaction, the reactants are mixed in the following order: water, PVP solution, silver nitrate solution, sulphite-bisulphite solution, and eventually the methylene glycol solution. After the reaction, the precipitates are collected by centrifugation, washed first with water, then with ethanol (redispersion was aided with gentle sonication). Reactions were carried out at room temperature (23 ± 1 °C). Reactions on a 10 mL-scale were conducted in 15 mL-glass vials, mixing with PTFE-coated magnetic stirrer bars (5x20 mm) rotating at 500 rpm.

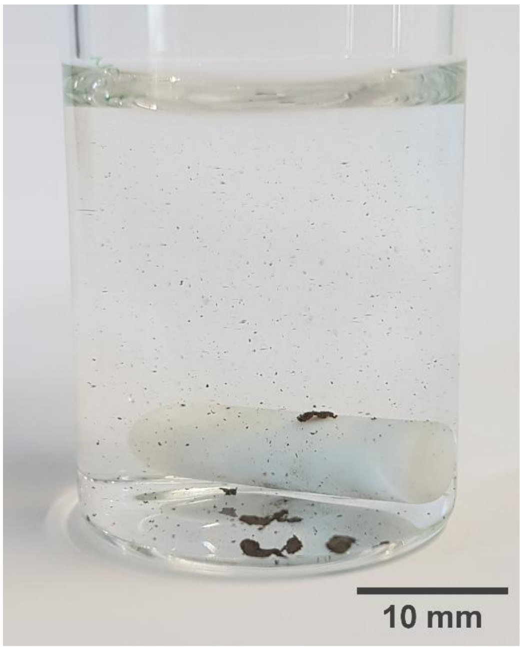

Figure S1. Picture of agglomerated silver nanoparticles in the reacted MGS-Ag(I) system without PVP.

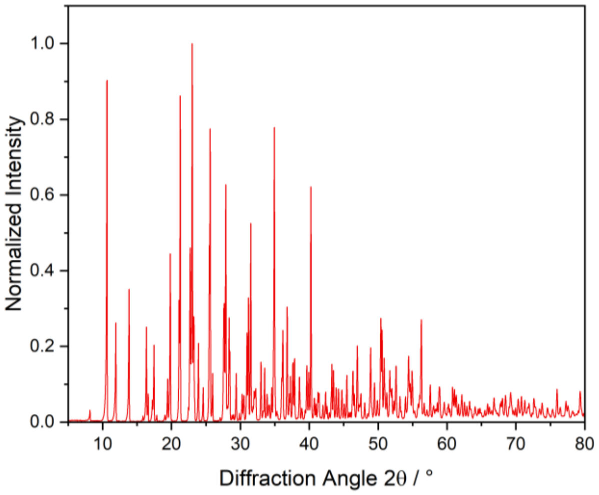

Figure S2. X-Ray diffractogram of sodium hydroxymethanesulphonate hydrate.

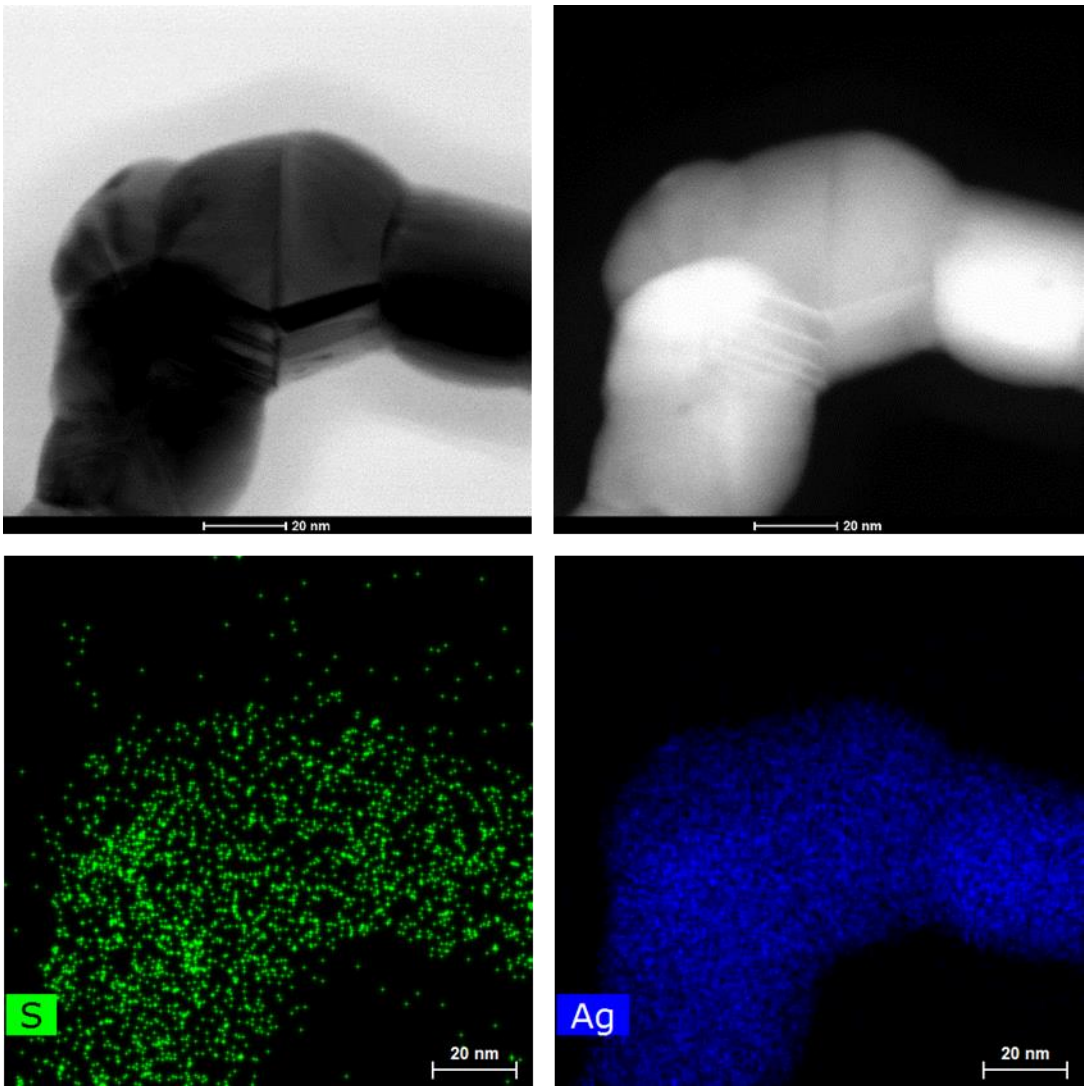

Figure S3. Scanning transmission electron micrographs (top left: bright field (BF), top right: high angle annular dark field (HAADF)) of the particles obtained from the MGS-Ag(I) system. Conditions: $[Ag(I)]_0$ = 0.5mM, $[MG]_0$ = 200mM. EDS mapping showed the presence of sulphur (bottom left) together with the silver nanoparticles, indicating the adsorption of hydroxymethanesulphonate on their surface.

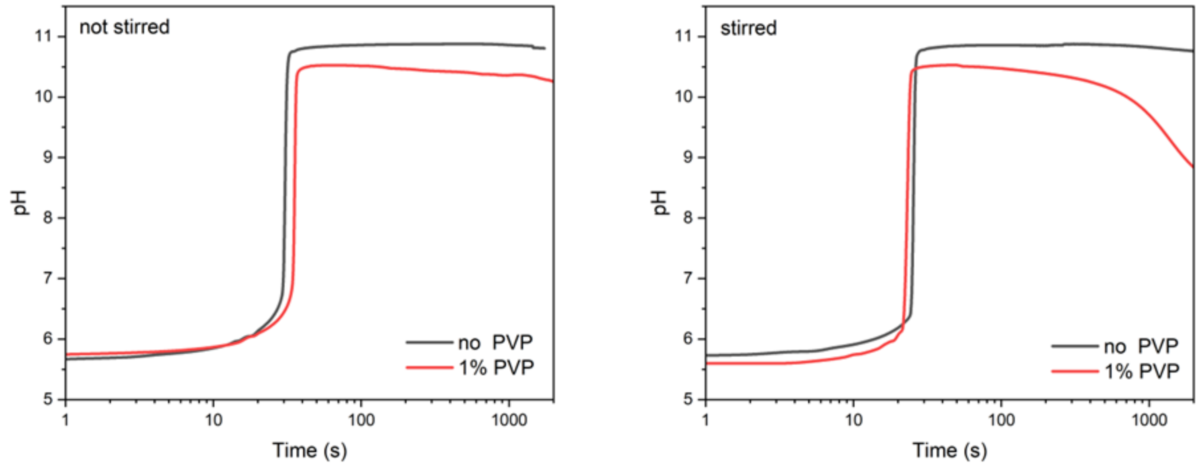

Figure S4. Comparison of the pH evolution over time for the MGS-Ag(I) system (without and with PVP) under stirred and unstirred conditions. $[Ag(I)]_0$ = 0.5mM, $[MG]_0$ = 200mM. The induction time is shorter under stirring, and the presence of PVP leads to a lower final pH.

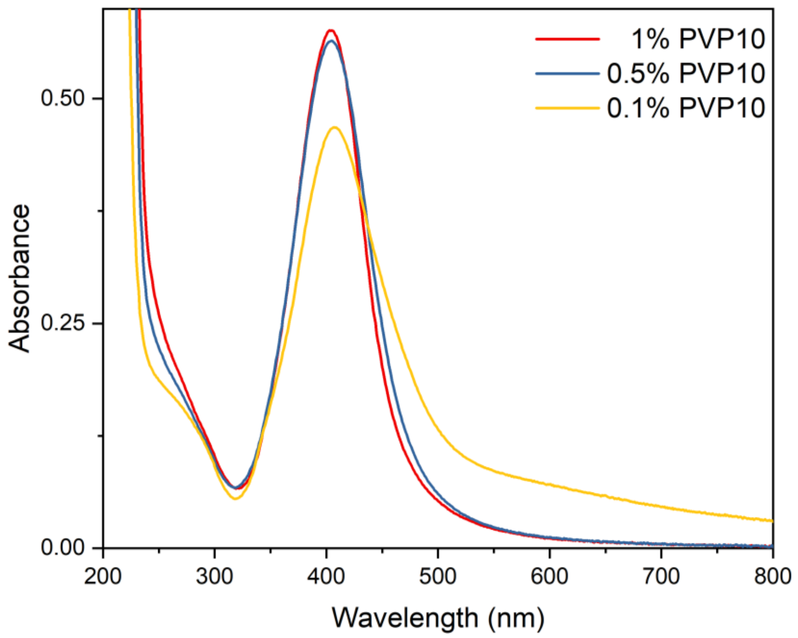

Figure S5. Comparison of the UV-vis spectra (with a characteristic SPR peak at ca. 400 nm) of the nanoparticle suspensions obtained with the MGS-Ag(I)-PVP system for varying amounts of PVP. Conditions: $[Ag(I)]_0$ = 0.5 mM, $[MG]_0$ = 200 mM. To avoid signal saturation, the as-synthesized samples were diluted 1:9 in volume with Milli-Q water. The presence of an absorption tail at 600-800 nm range for the sample obtained with 0.1% PVP suggested an insufficient stabilisation of the nanoparticles. By contrast, the intense and relatively narrow SPR peak, with negligible absorption at long wavelengths, of the samples obtained with 0.5% and 1% PVP are indicative of well-dispersed, spherical, and small (<100 nm in diameter) silver nanoparticles.

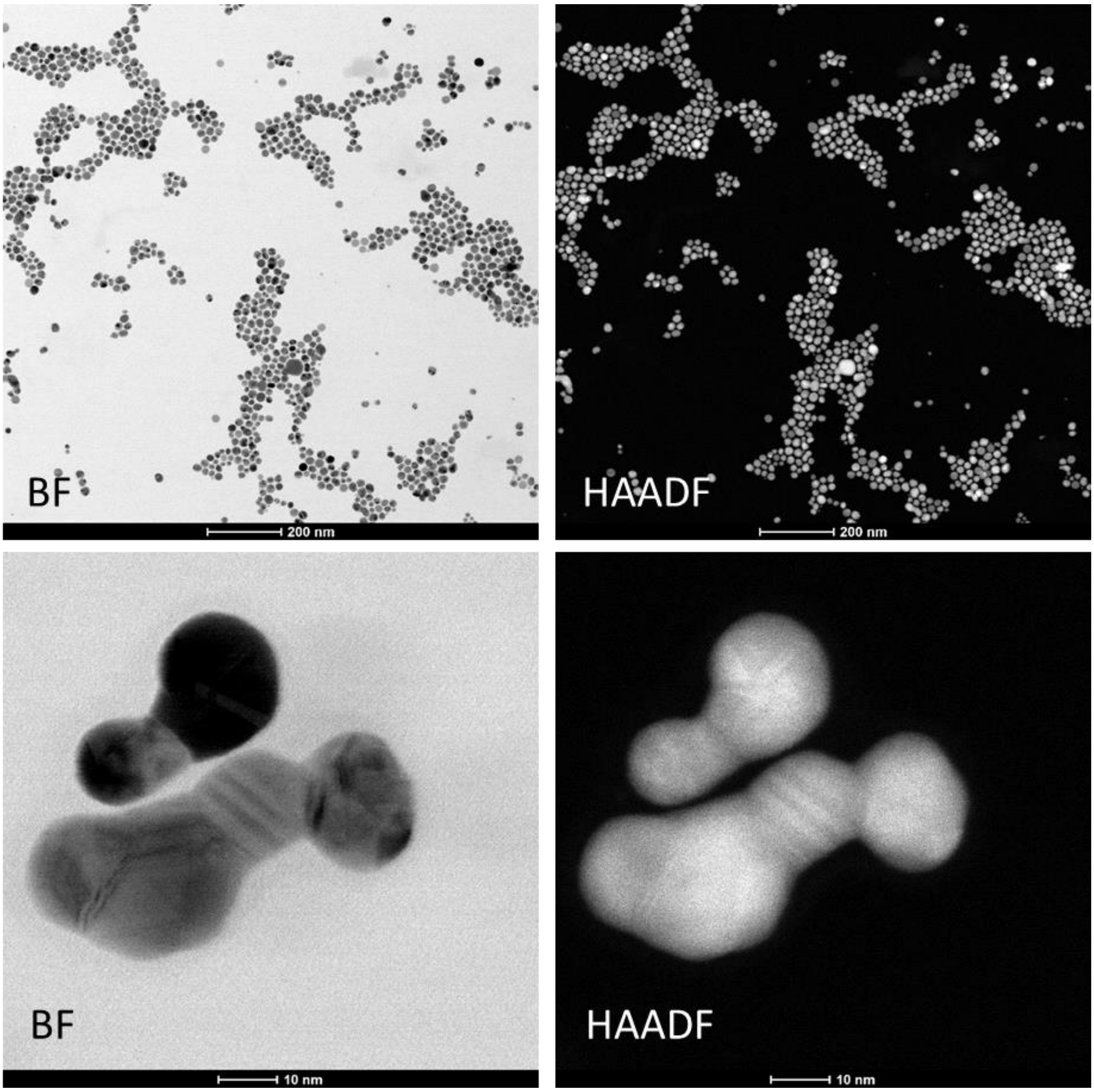

Figure S6. STEM micrographs of particles obtained from the MGS-Ag(I) system with 0.5% PVP. Conditions: $[Ag(I)]_0$ = 0.5 mM, $[MG]_0$ = 200 mM.

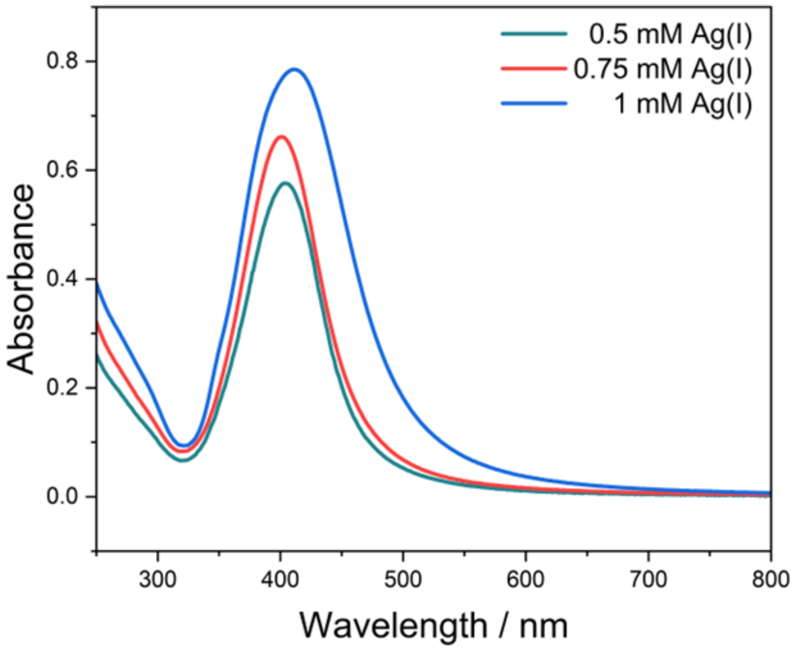

Figure S7. Comparison of the UV-vis spectra of the nanoparticle suspensions obtained with the MGS-Ag(I)-PVP system for varying $[Ag]_0$. To avoid the saturation of the light absorption signal, the as-synthesized samples were diluted 1:9 in volume with Milli-Q water. Conditions: $[MG]_0$ = 200 mM, PVP 1%.

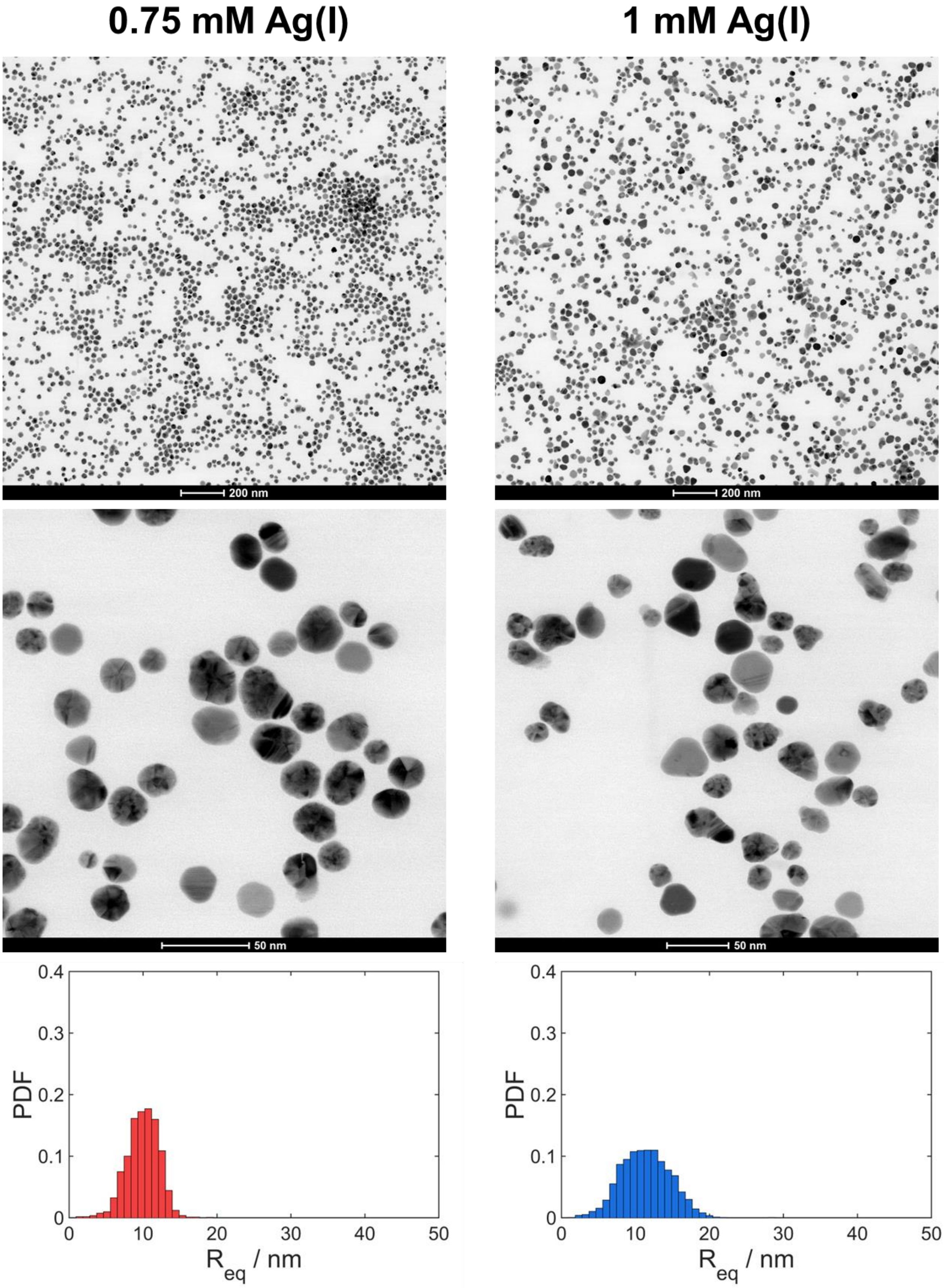

Figure S8. STEM micrographs and size distributions of the particles obtained from the MGS-Ag(I)-PVP system for varying $[Ag]_0$. Conditions: $[MG]_0$ = 200 mM, 1% PVP.

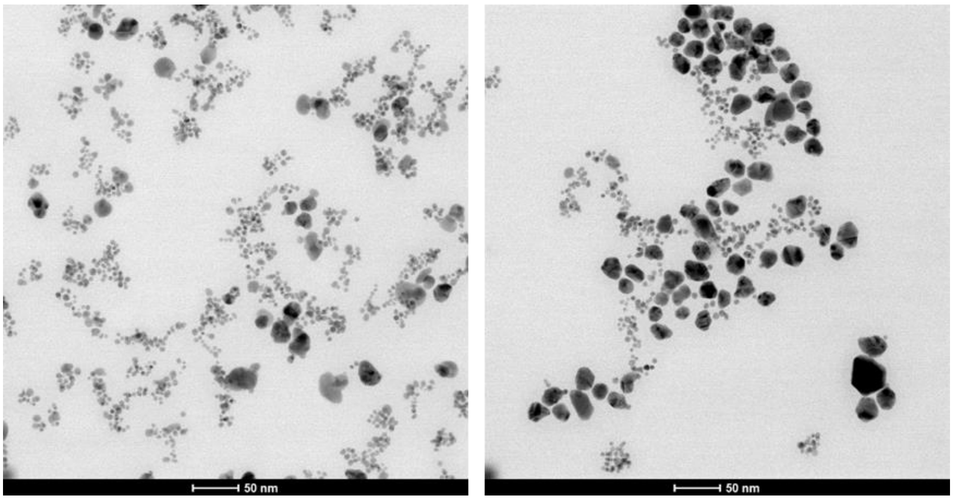

Figure S9. BF-STEM micrographs of a control sample obtained by adding base to a solution of Ag(I) containing PVP. Conditions: $[Ag(I)]_0$ = 0.5 mM, 1% PVP, $[NaOH]_0$ = 5mM.

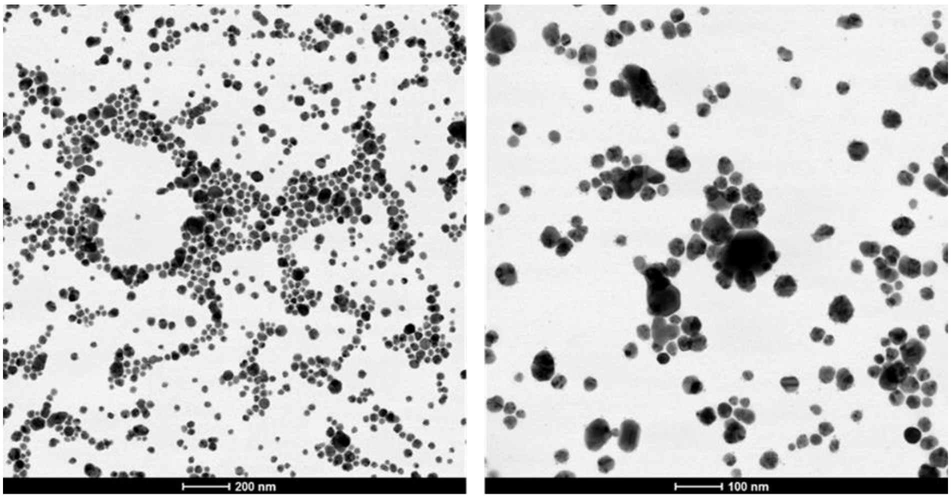

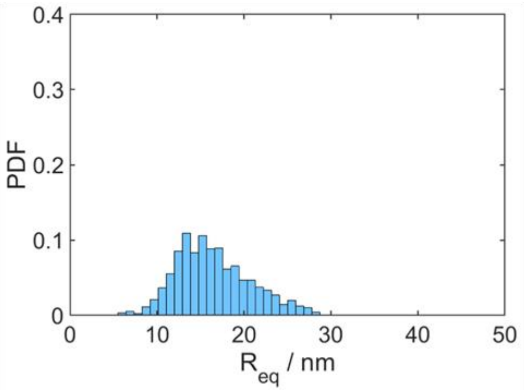

Figure S10. BF-STEM micrographs and size distribution analysis of a control sample obtained by adding base to a solution of Ag(I) containing PVP, in presence of MG. Conditions: $[Ag(I)]_0$ = 0.5 mM, $[MG]_0$ = 200mM, 1% PVP, $[NaOH]_0$ = 5mM.

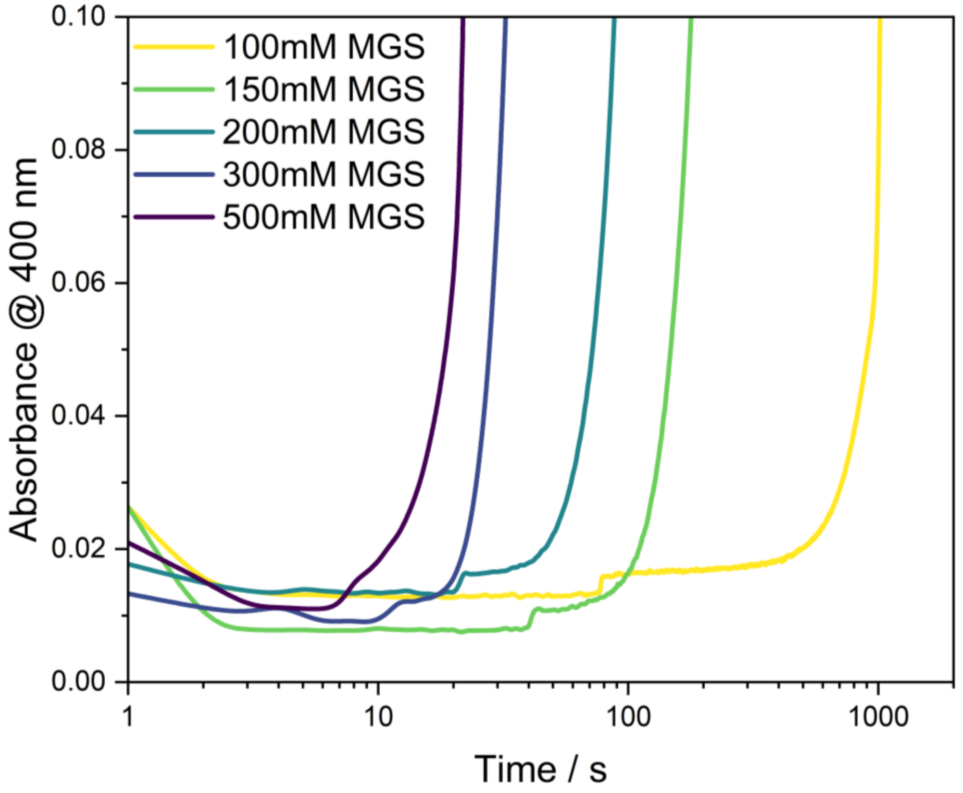

Figure S11. Zoomed-in view of the evolution of absorbance in the MGS-Ag(I)-PVP system as a function of $[MG]_0$, showing a distinct and sudden increase in absorbance in correspondence of the pH increase (see manuscript, **Figure 2**).

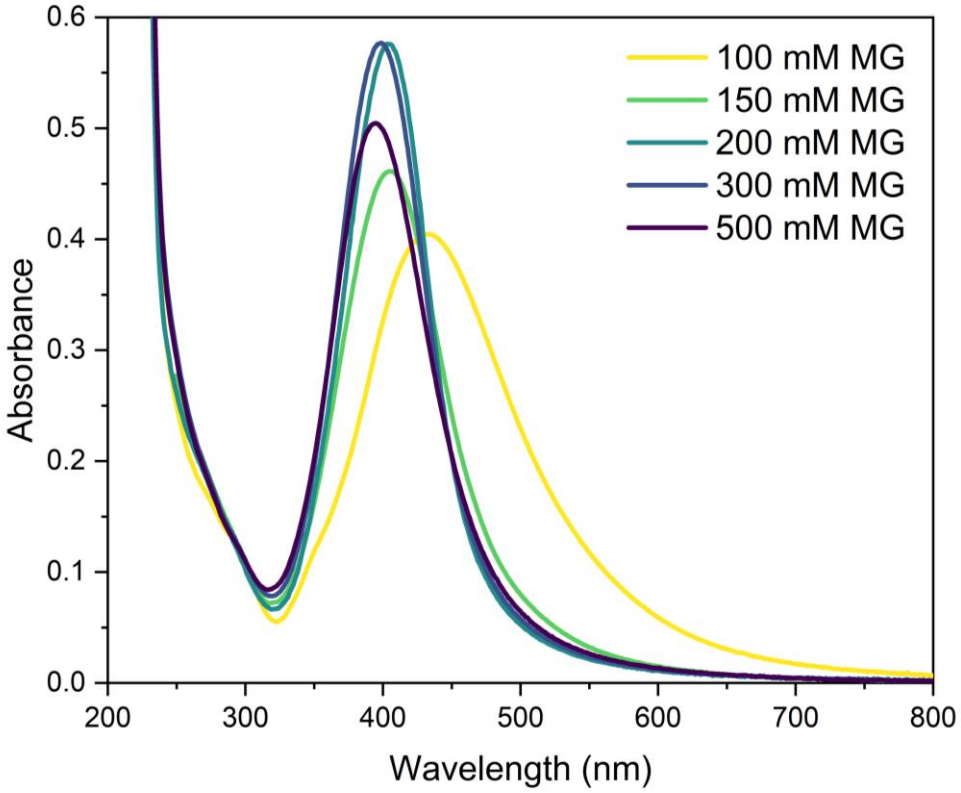

Figure S12. UV-Vis spectra of the samples obtained with the MGS-Ag(I)-PVP system as a function of $[MG]_0$. To avoid the saturation of the light absorption signal, the as-synthesized samples were diluted 1:9 in volume with Milli-Q water. Conditions: $[Ag(I)]_0$ = 0.5 mM, 1% (w/v) PVP.

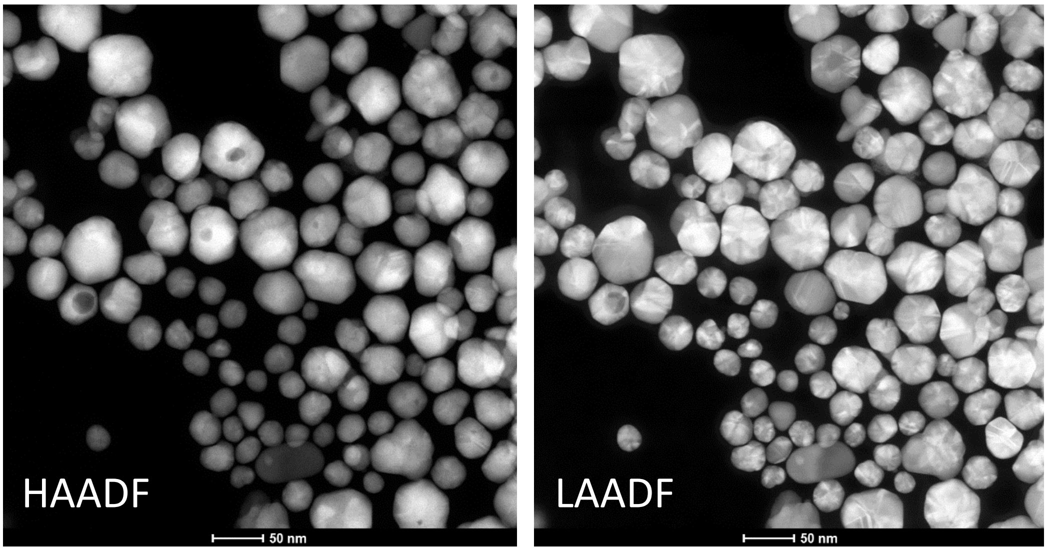

Figure S13. HAADF and LAADF STEM micrographs of particles obtained with the MGS-Ag(I) in presence of 1% PVP. Conditions: $[Ag(I)]_0$ = 0.5 mM, $[MG]_0$ = 100 mM.

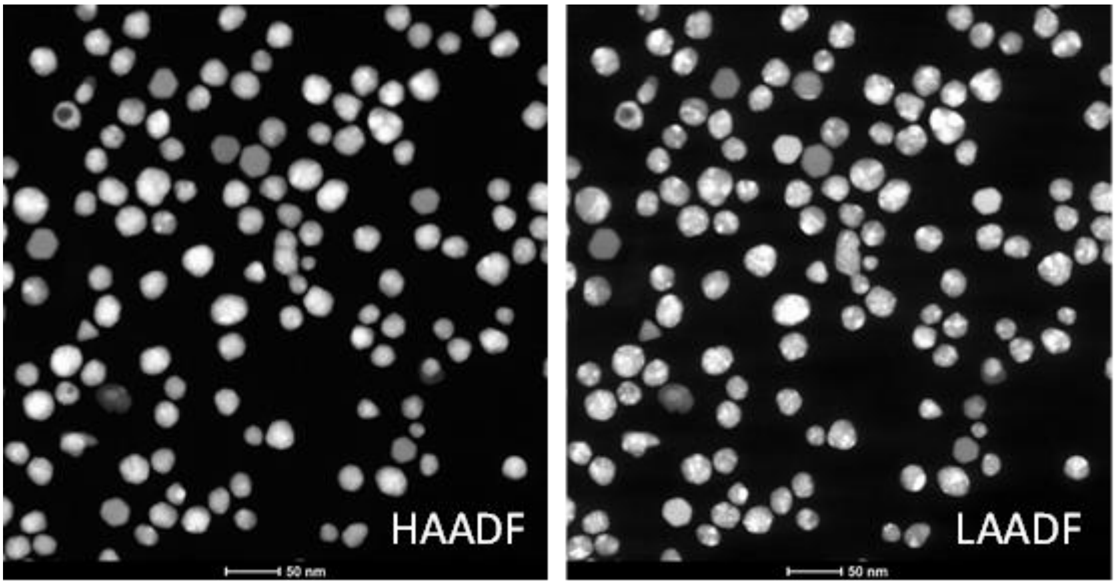

Figure S14. HAADF and LAADF STEM micrographs of particles obtained with the MGS-Ag(I) in presence of 1% PVP. Conditions: $[Ag(I)]_0$ = 0.5 mM, $[MG]_0$ = 150 mM.

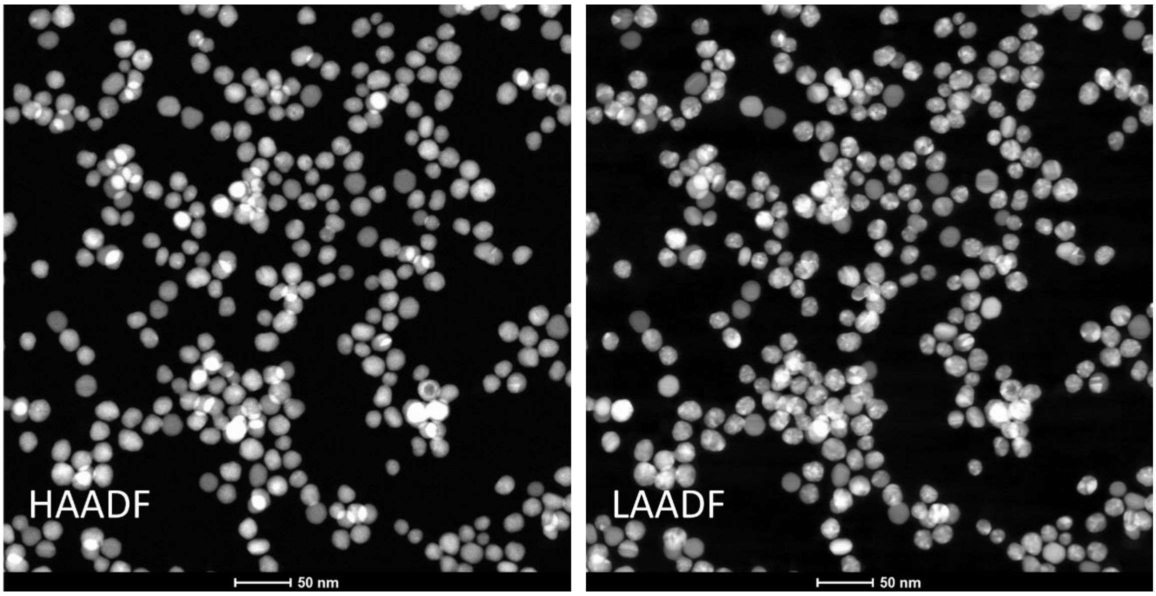

Figure S15. HAADF and LAADF STEM micrographs of particles obtained with the MGS-Ag(I) in presence of 1% PVP. Conditions: $[Ag(I)]_0$ = 0.5 mM, $[MG]_0$ = 200 mM.

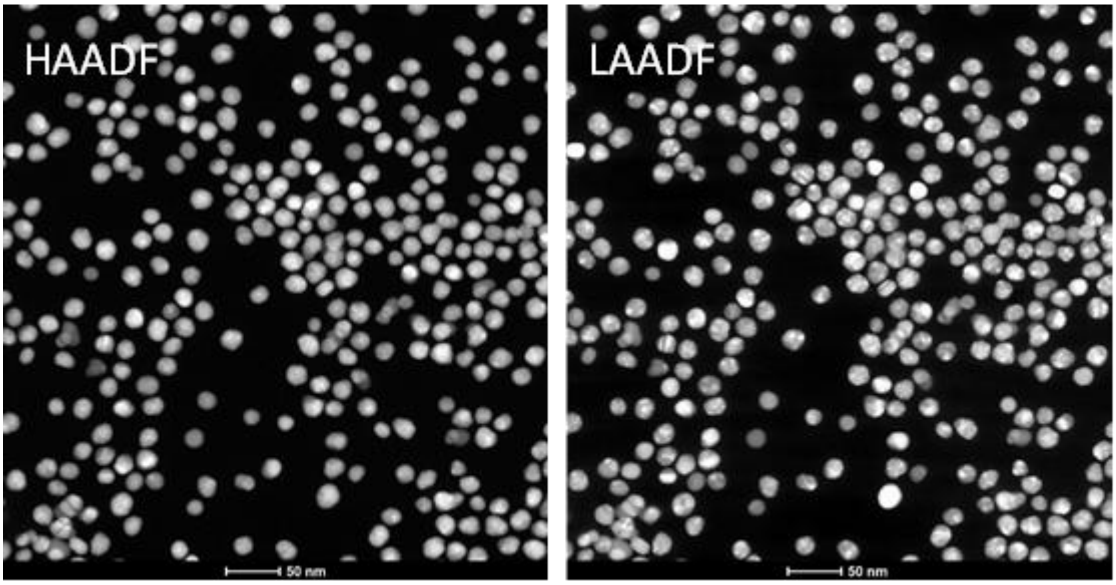

Figure S16. HAADF and LAADF STEM micrographs of particles obtained with the MGS-Ag(I) in presence of 1% PVP. Conditions: $[Ag(I)]_0$ = 0.5 mM, $[MG]_0$ = 300 mM.

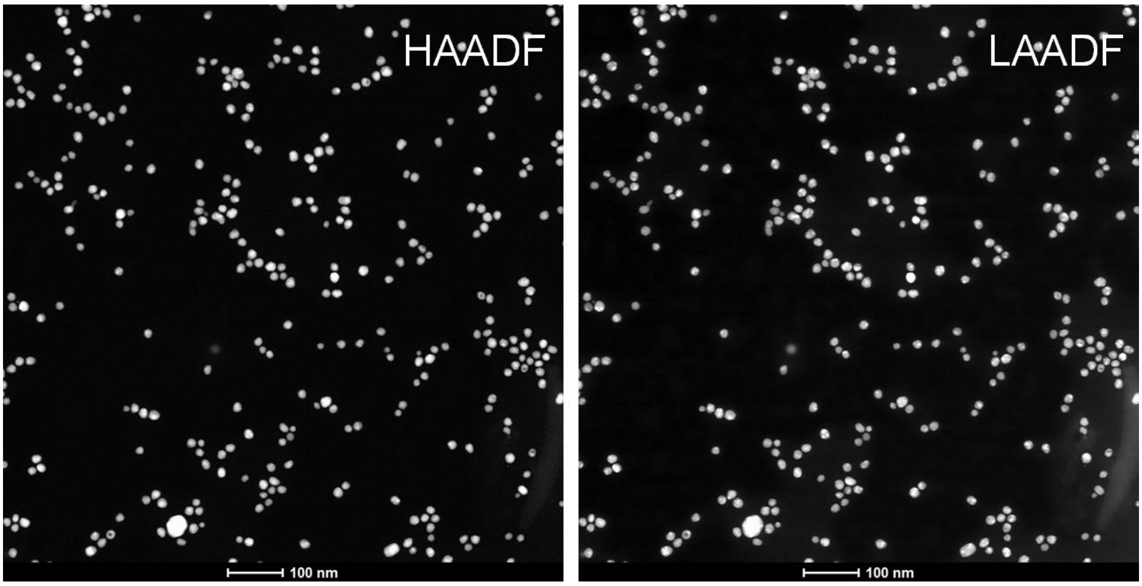

Figure S17. HAADF and LAADF STEM micrographs of particles obtained with the MGS-Ag(I) in presence of 1% PVP. Conditions: $[Ag(I)]_0$ = 0.5 mM, $[MG]_0$ = 500 mM.

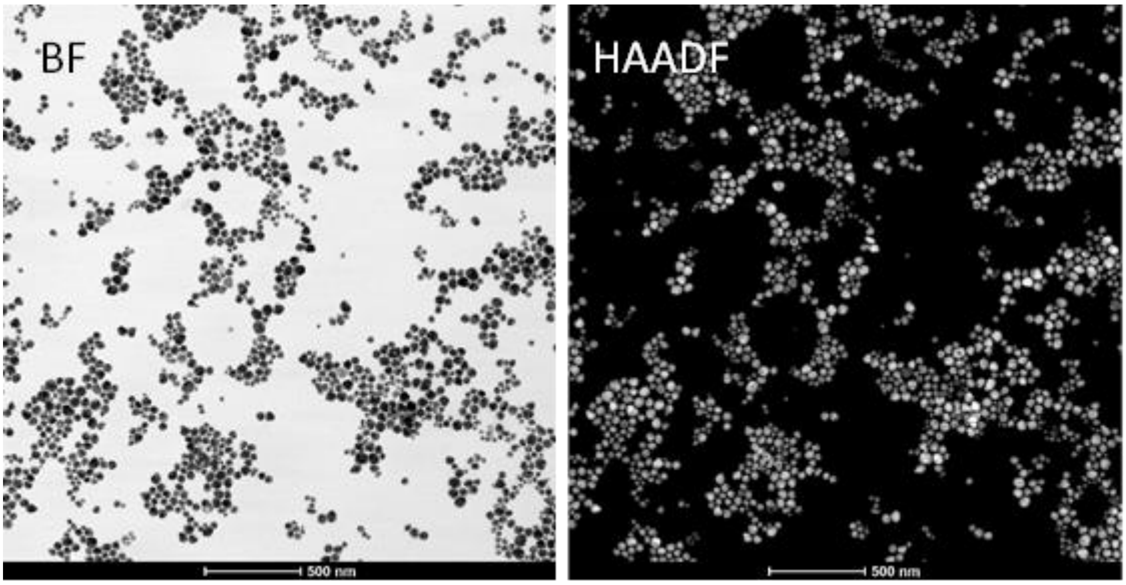

Figure S18. BF and HAADF STEM micrographs of particles obtained with the MGS-Ag(I) in presence of 1% PVP. Conditions: $[Ag(I)]_0$ = 0.5 mM, $[MG]_0$ = 100 mM.

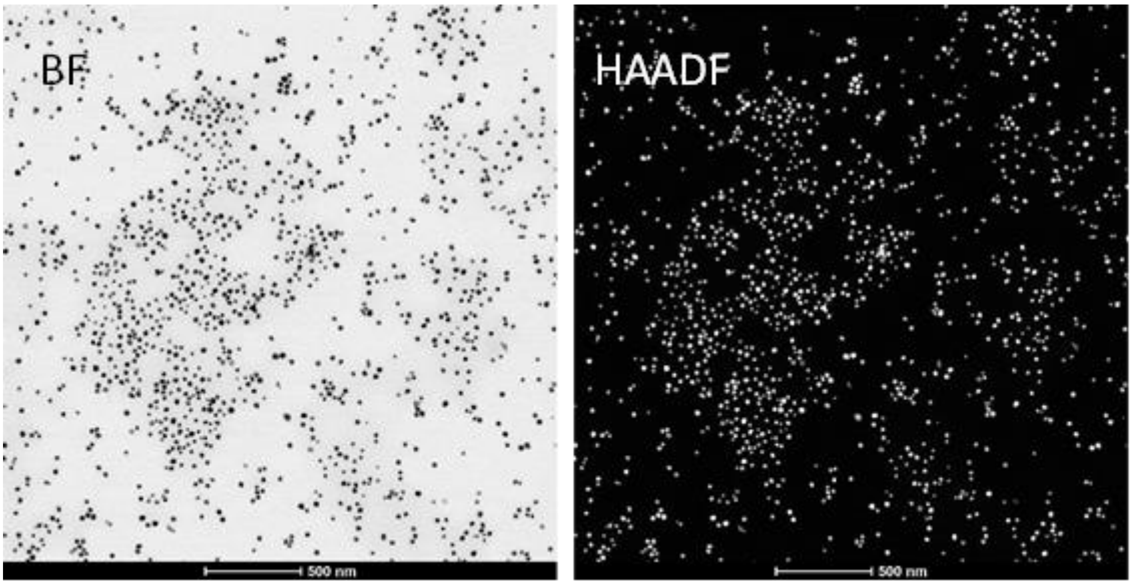

Figure S19. BF and HAADF STEM micrographs of particles obtained with the MGS-Ag(I) in presence of 1% PVP. Conditions: $[Ag(I)]_0$ = 0.5 mM, $[MG]_0$ = 150 mM.

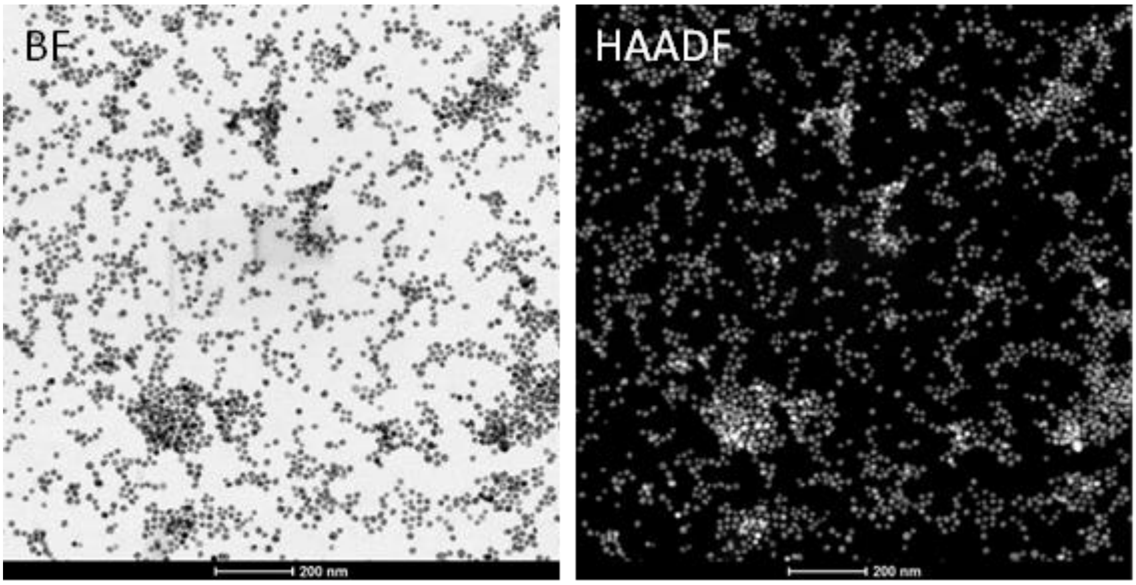

Figure S20. BF and HAADF STEM micrographs of particles obtained with the MGS-Ag(I) in presence of 1% PVP. Conditions: $[Ag(I)]_0$ = 0.5 mM, $[MG]_0$ = 200 mM.

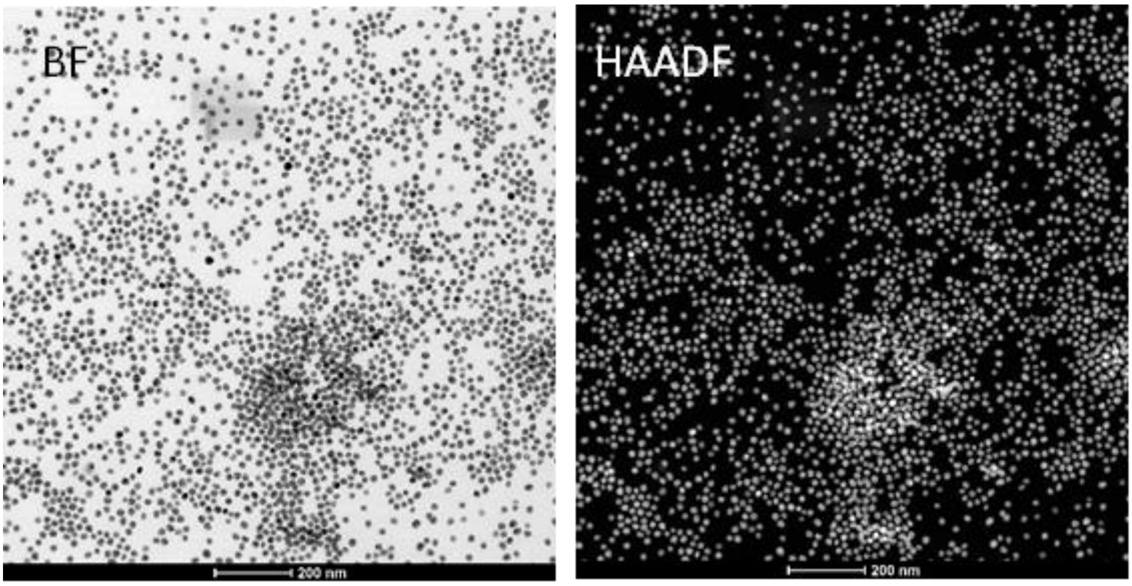

Figure S21. BF and HAADF STEM micrographs of particles obtained with the MGS-Ag(I) in presence of 1% PVP. Conditions: $[Ag(I)]_0$ = 0.5 mM, $[MG]_0$ = 300 mM.

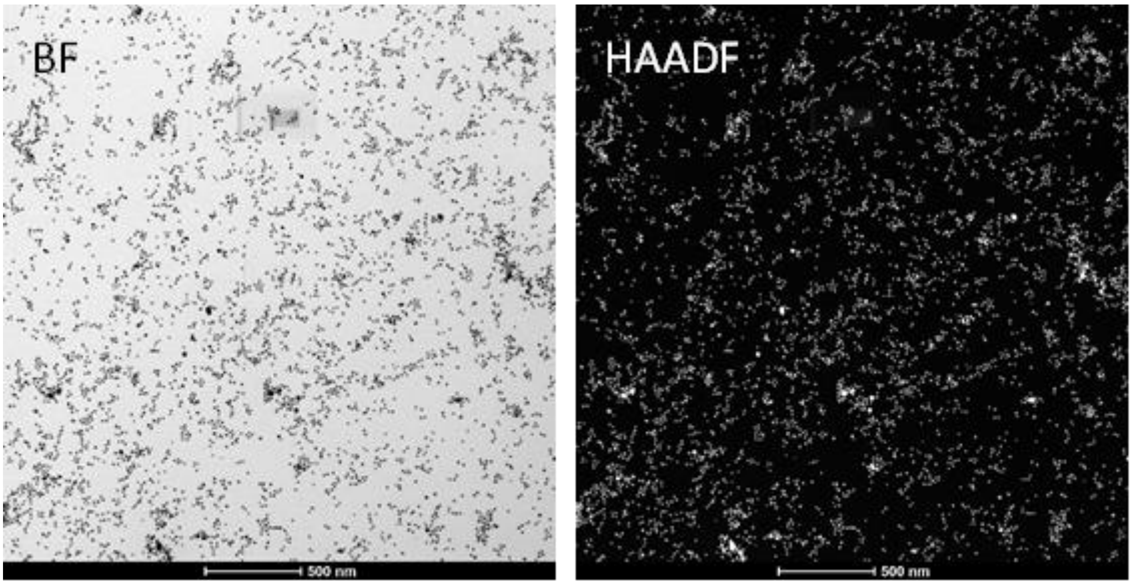

Figure S22. BF and HAADF STEM micrographs of particles obtained with the MGS-Ag(I) in presence of 1% PVP. Conditions: $[Ag(I)]_0$ = 0.5 mM, $[MG]_0$ = 500 mM.

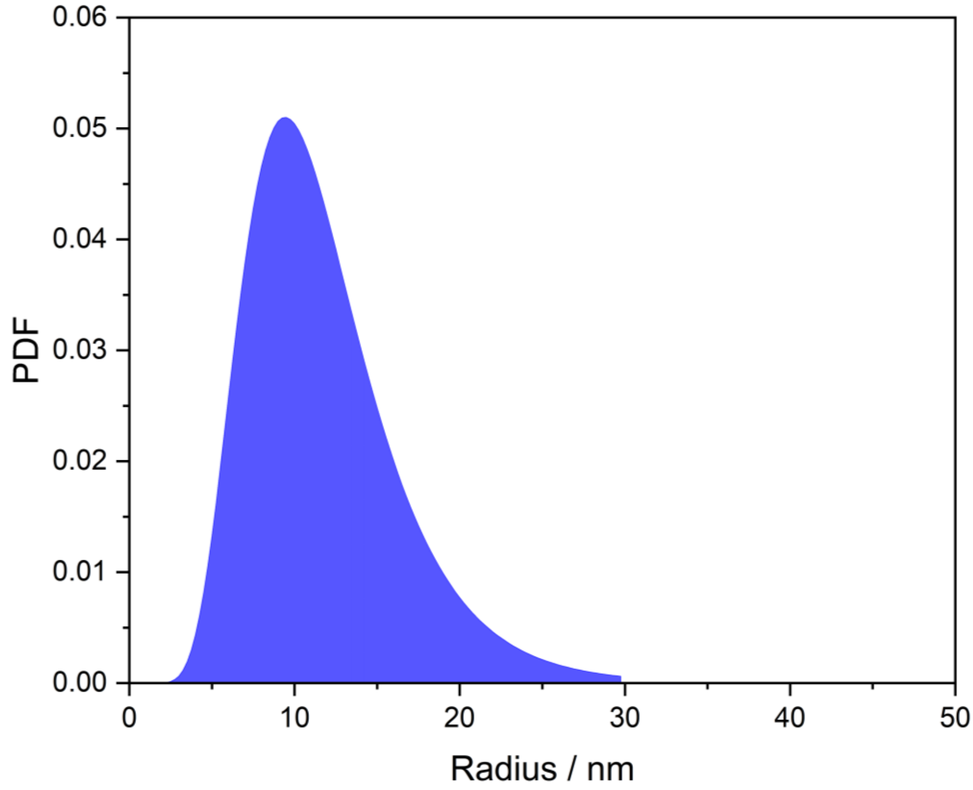

Figure S23. Size distribution from SAXS of a control sample obtained with $[Ag]_0$ = 0.5 mM, $[MG]_0$ = 200 mM, $[NaOH]_0$ = 5 mM, 1% (w/v) PVP.

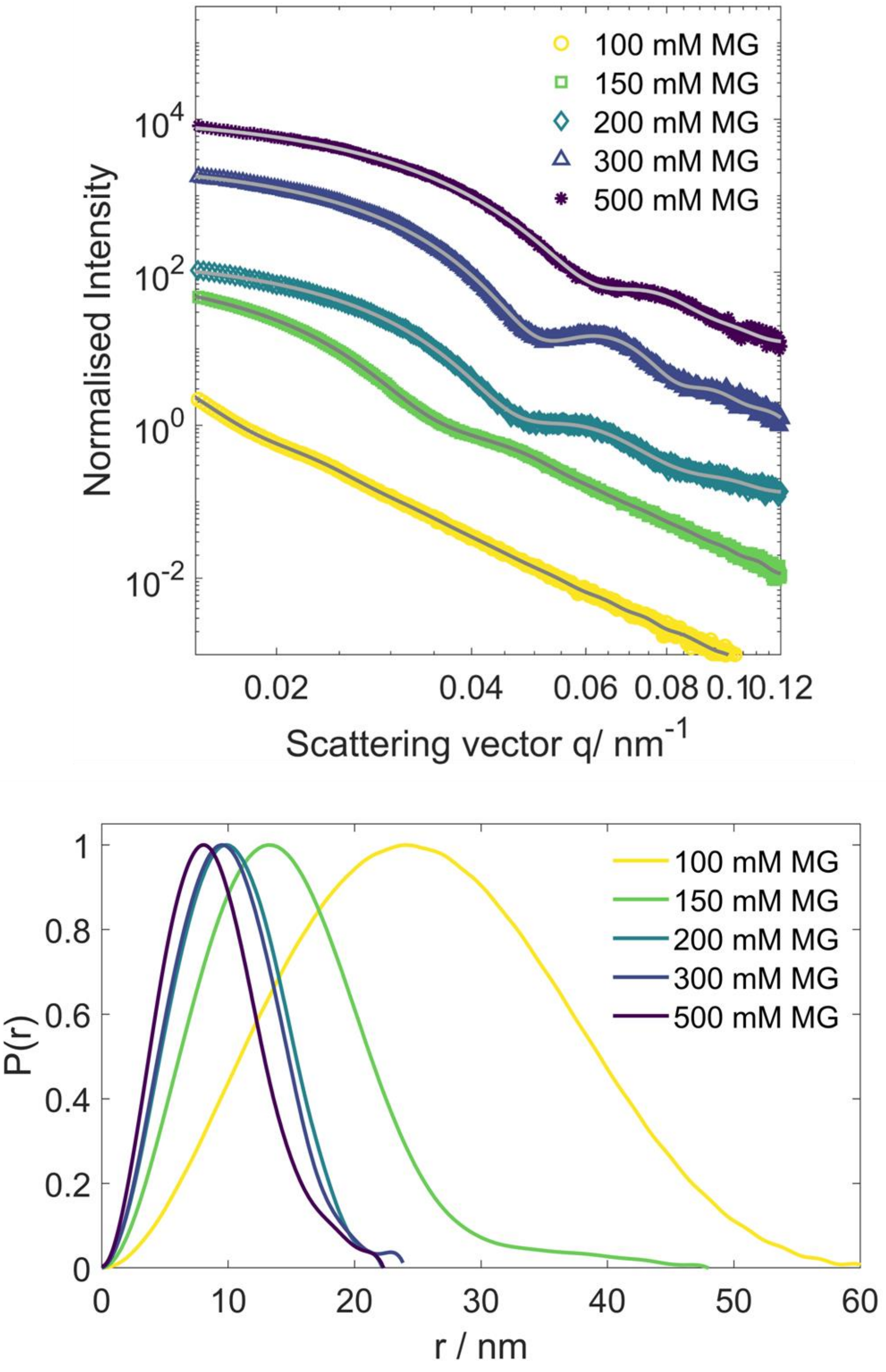

Figure S24. (top) Experimental SAXS data and corresponding fit for (bottom) the PDDF.

References

[1] Schindelin, J., Arganda-Carreras, I., Frise, E., Kaynig, V., Longair, M., Pietzsch, T., Preibisch, S., Rueden, C., Saalfeld, S., Schmid, B., Tinevez, J.-Y., White, D. J., Hartenstein, V., Eliceiri, K., Tomancak, P.,Cardona, A. **Fiji: an open-source platform for biological-image analysis.** *Nature Methods* **2012**, *9*, 676-682.

[2] Vincent, L. and Soille, P., **Watersheds in digital spaces: An efficient algorithm based on immersion simulations.** *IEEE PAMI* **1991**, *13*, 583-598.

[3] Xenocs, **XSACT: X-ray Scattering Analysis and Calculation Tool.** xsact.xenocs.com, **2021**. SAXS & WAXS data analysis software — Version 2.6.

[4] Manalastas-Cantos, K., Konarev, P.V., Hajizadeh, N.R., Kikhney, A.G., Petoukhov, M.V., Molodenskiy, D.S., Panjkovich, A., Mertens, H.D.T., Gruzinov, A., Borges, C., Jeffries, C.M., Svergun, D.I., Franke, D. (2021) **ATSAS 3.0: expanded functionality and new tools for small-angle scattering data analysis.** *J. Appl. Cryst.* **2021**, *54*, 343-355.
